# The Role of Branched-Chain Amino Acids and Branched-Chain α-Keto Acid Dehydrogenase Kinase in Metabolic Disorders

**DOI:** 10.3389/fnut.2022.932670

**Published:** 2022-07-18

**Authors:** Chuang Du, Wen-Jie Liu, Jing Yang, Shan-Shan Zhao, Hui-Xin Liu

**Affiliations:** ^1^Institute of Life Sciences, China Medical University, Shenyang, China; ^2^Health Sciences Institute, China Medical University, Shenyang, China; ^3^Liaoning Key Laboratory of Obesity and Glucose/Lipid Associated Metabolic Diseases, China Medical University, Shenyang, China; ^4^Department of Gastroenterology, Shengjing Hospital of China Medical University, Shenyang, China; ^5^Department of Gynecology, Cancer Hospital of China Medical University, Liaoning Cancer Hospital & Institute, Shenyang, China

**Keywords:** branched-chain amino acids, branched-chain α-keto acid dehydrogenase kinase, metabolic disorders, microbiota, MALDI-MSI

## Abstract

Branched-chain amino acids (BCAAs), composed of leucine, isoleucine, and valine, are important essential amino acids in human physiology. Decades of studies have revealed their roles in protein synthesis, regulating neurotransmitter synthesis, and the mechanistic target of rapamycin (mTOR). BCAAs are found to be related to many metabolic disorders, such as insulin resistance, obesity, and heart failure. Also, many diseases are related to the alteration of the BCAA catabolism enzyme branched-chain α-keto acid dehydrogenase kinase (BCKDK), including maple syrup urine disease, human autism with epilepsy, and so on. In this review, diseases and the corresponding therapies are discussed after the introduction of the catabolism and detection methods of BCAAs and BCKDK. Also, the interaction between microbiota and BCAAs is highlighted.

## Introduction

Branched-chain amino acids (BCAAs) account for about 35% of essential amino acids in most mammals, which cannot be synthesized by mammals themselves, and the functional R group makes them vital components of most proteins (1). BCAAs, composed of leucine, isoleucine, and valine, are not synthesized in animals but in plants, bacteria, and fungi. They have become popular study subjects since their discovery in the mid-nineteenth century. All BCAA members play important roles in nutrient sensing and cellular signaling, especially leucine, which acts directly by activating the mammalian/mechanistic targets of rapamycin complex 1 (mTORC1) (2). BCAAs have been studied due to their beneficial role in promoting protein synthesis in muscle during physical training and under conditions of negative energy balance, such as syndromes of cachexia and aging. Moreover, continuous accumulation in BCAAs from circulation is reported in patients with insulin resistance, obesity, type 2 diabetes, as well as heart diseases (3).

## Branched-Chain Amino Acid Metabolism

**FIGURE 1:**
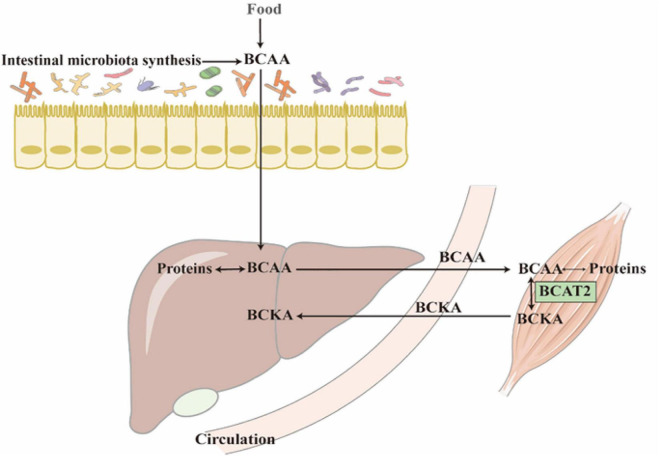
Most of the BCAAs come from protein decomposition and food absorption. The reversible transamination reaction of BCAAs catabolism mostly occurs in skeletal muscle. After BCKAs are released back into circulation, most of them are oxidatively decarboxylated to acyl-CoA in the liver.

**FIGURE 2:**
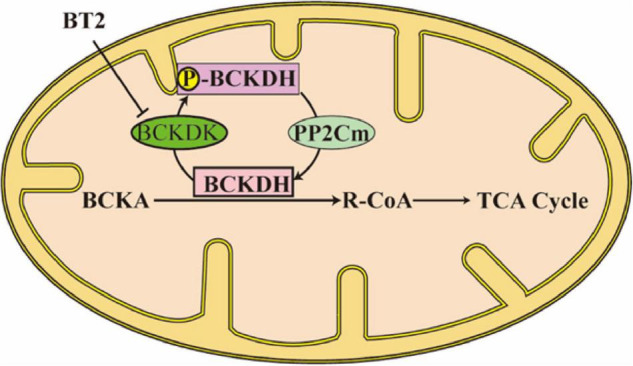
A brief schematic of BCKA catabolism, and the enzymes that regulate this process.

In BCAAs metabolism, the first step is BCAAs’ transforming into branched-chain α-ketoacids (BCKAs) under the catalyzation of branched-chain aminotransferases (BCATs; Figure 1). The corresponding BCKAs of leucine, isoleucine, and valine are α-ketoisocaproate (KIC), α-keto-β-methylvalerate (KMV), and α-ketoisovalerate (KIV) (4). Two different genes which encode BCATs are responsible for this process: BCAT1 is mostly expressed in the brain and encodes a cytoplasmic protein, at the same time, BCAT2 is widely expressed and encodes a mitochondrial protein (1). This step is a reversible transamination reaction, that largely occurs in skeletal muscle. After BCKAs are released back into circulation, they are oxidatively decarboxylated to acyl-CoA mainly in the liver. The branched-chain α keto-acid dehydrogenase (BCKDH) complex is an important enzyme that catalyzes this irreversible and rate-controlling process in BCAA catabolism. Although the liver and other tissues (such as adipose and muscle) all possess BCKDH and are able to carry out the catabolism of BCKAs, the liver is reported as the highest metabolic efficiency organ in the body in the oxidative decarboxylation of BCKAs (5). BCKAs covalently bind to a coenzyme A group and lose CO_2_ in an oxidative decarboxylation process catalyzed by BCKDH (1). The BCKDH complex consists of three parts, namely, E1, E2, and E3. BCKDH converts KIC to isovaleryl-CoA (IV-CoA), KMV to α-methylbutyryl-CoA (MB-CoA), and KIV to isobutyryl-CoA (IB-CoA). *BCKDHA* and *BCKDHB* genes encode E1, which exists as an α2/β2 heterotetramer, and its role is a thiamin-dependent decarboxylase. The *DBT* gene encodes E2, which acts as a lipoate-dependent dihydrolipoyl transacylase, transferring the acyl groups to coenzyme A. The *DLD* gene encodes E3, which is a FAD-dependent dihydrolipoyl dehydrogenase and functions in transferring the released electrons to NAD^+^ (1). BCKDH kinase (BCKDK) suppresses the activity of BCKDH by adding phosphate to three residues of BCKDHA. BCKAs especially the KIC allosterically regulate BCKDK. If the BCKAs are at high concentration, BCKAs will inhibit the activity of BCKDK, preventing BCAAs from running out when they are at low concentration (6). However, BCKDK also seems to be regulated by the BCKDH complex, the details of the mechanism need to be studied (6). The dephosphorylation process which activates the BCKDH complex is catalyzed by mitochondrial phosphatase 2C (PP2Cm). After the decarboxylation of BCKAs catalyzed by BCKDH, the following catabolism step is similar to the oxidation of fatty acid, every reaction is unique to the three BCAAs, and the mitochondrial matrix is the only site these reactions take place (Figure 2). In the end, the carbons from BCAA catabolism are either released as CO_2_ or enter the tricarboxylic acid (TCA) cycle (1). Except for complete oxidation, the BCAA intermediates also underwent degradation through gluconeogenesis, lipogenesis, ketogenesis, or cholesterol synthesis pathway (7).

## Detection Methods of Branched-Chain Amino Acids and Branched-Chain α-Keto Acid Dehydrogenase Kinase

Nowadays, there is a great progress in the detection of amino acids at very low concentrations in complex matrixes, including liquid chromatography-mass spectrometry (LC-MS) and capillary electrophoresis-mass spectrometry (CE-MS) (6, 8, 9). Liquid chromatography and tandem mass spectrometry appear to be the major methods for detecting plasma BCAAs nowadays (10–13). Also, there are other improved LC-MS methods, including isotope dilution liquid chromatography tandem mass spectrometry (ID-LC/MS/MS) and high-performance liquid chromatography with electrospray ionization mass spectrometry (LC-ESI-MS) (14, 15). Due to the significant biological role that BCAAs and BCKAs play in metabolism, being able to measure them at the same time has become an important topic. A liquid chromatography-electrospray ionization-tandem mass spectrometry (LC-ESI±-MS/MS) method makes it possible to investigate BCAAs accompanied by BCKAs in human serum simultaneously (10). The CE-MS method (9, 16–18) is a valuable alternative for detecting BCAA concentrations. This method is widely used in the quantification of BCAAs in fluids and food, also, it has a wide application in pharmaceutical quality control (16).

Recently, a novel method of matrix-assisted laser desorption ionization-mass spectrometry imaging (MALDI-MSI) has been used to analyze BCAAs localization in tissues, especially in the brain. Many researches have demonstrated that the derivatization reagents such as 4-(anthracen-9-yl)-2-fluoro-1-methylpyridin-1-ium iodide (FMP-10), 2,4-diphenyl-pyranylium tetrafluoroborate (DPP), 2,3-diphenyl-pyranylium tetrafluoroborate (DPP-TFB), and 2,4,6-trimethylpyrylium (TMP) can promote amino metabolites detection in MALDI-MSI (19–21). This technique can simultaneously locate and quantify BCAAs in tissue sections, giving researchers credible information when studying the spatial distribution and quantity of BCAAs. Also, this technique makes it possible to study the impact of medication intervention on BCAAs.

The detection methods for BCKDK are quite simple. Real-time quantitative PCR, western blot, and immunohistochemical assay are used to analyze the expression of BCKDK. The activity of BCKDH also represents BCKDK function, and it can be measured by scintigraphy (22). The phosphorylation rate of BCKDH represents BCKDK function as well. At the same time, the function of BCKDK in glucose metabolism can be measured by the uptake amount of glucose, the production level of lactate, the oxygen consumption rate in the cell, the extracellular acidification rate, and reactive oxygen species (23, 24).

## Microbiota and Branched-Chain Amino Acid-Associated Metabolic Disorder

Food nutrients are regarded as the main nutrition supply for humans and other mammals. However, the gut microbiota has been reported to play an important role in modulating the utilization of numerous necessary nutrition for the host. Increasing evidence shows that obesity and many metabolic disorders may be related to the circulating BCAA pool, which can be affected by gut microbiota (25). Given the same protein proportion in high-fat diets (HFD) and normal control diets (NCD), bacterial metabolism is considered to be the source of high levels of serum BCAAs in HFD. In diet-induced obese (DIO) mice which are treated with luffa administration, the relative abundances of *g-Enterortabdus*, *g-Eubacterium-xylanophilum*-group, and *g-Butyricicoccus* were found to be increased in 16S rRNA gene sequencing (26). Also, in dietary luffa-treated DIO mice, the mRNA expression of the enzymes that catalyze BCAA the catabolism to decrease BCAA levels is upregulated in tissues such as the liver, adipose, and colon (26). Gut microbiota is altered in patients with stable heart failure, through the use of metagenomic analysis. Patients with heart failure have lower plasma essential amino acid levels than healthy controls. The lack of *Eubacterium* and *Prevotella* leads to the decreased biosynthesis of essential amino acids (especially BCAAs and histidine) in patients with heart failure, on account of the reduced abundance of microbial genes related to essential amino acid biosynthesis (27). *Prevotella-copri* is lipopolysaccharide-producing bacteria, making it related to low-grade inflammation and the synthesis of BCAAs (28).

Branched chain amino acids have potential effects on lipid composition. BCAAs metabolize into acetyl coenzyme A and propionyl coenzyme A, which are substrates for lipid synthesis. Excess carbon produced by catabolism of BCAA may lead to an increase in the rate of adipogenesis (29). Research has reported that fat accumulation in the liver was reduced after BCAAs supplementation in a rat experiment. The supplementation of BCAA will lead to the proliferation of *R. flavefaciens* which synthesized and released acetic acid into the portal vein, so that the expression of fatty-acid synthase (FAS) and acetyl-CoA carboxylase (ACC) in the liver is downregulated, reducing the occurrence of non-alcoholic fatty liver disease (NAFLD) (30).

Generally, individuals with obesity are prone to having gut microbiota dysbiosis with higher levels of circulating BCAAs and BCKAs compared with lean individuals. It is reported that gavage with *Bacteroides spp*. reduced the defects in BCAA catabolism of brown adipose tissue (BAT) caused by obesity and protected mice from obesity. The supplementation of *Bacteroides spp.* alters gut microbiota composition and reduces the levels of BCAA and BCKA in BAT, proving the beneficial effect of *Bacteroides spp.* in the catabolism of BCAA in BAT (31). Many studies that are targeted at microbiota intervention have suggested that regulating the BCAAs can be achieved by changing the gut microbiota, thus showing a promising therapeutic prospect. Some key findings focused on BCAAs and microbiota are summarized in Table 1.

**TABLE 1:** Summary of the interaction between microbiota and BCAA levels in different experimental settings from the recent studies.

Model	Treatment	Finding	Microbiome composition	Changes of BCAA	Refs
Mice	HFD, luffa gavage	Dietary luffa reduced higher circulating BCAA and upregulated BCAA catabolizing enzymes. In germ-free-mimic mice, dietary *luffa* did not influence BCAA catabolism when mice are fed HFD and formed DIO.	Decreased relative abundances of *g_Enterortabdus*, *g_Eubacterium_xylanophilum_group* and *g_Butyricicoccus*.	Decreased circulating BCAA levels.	(26)
Human	Stable heart failure patients	The depletion of *Eubacterium* and *Prevotella* cause the decreased abundance of microbial genes which are related to BCAAs biosynthesis in the heart failure patients.	Decreased *Eubacterium* and *Prevotella.*	Decreased circulating BCAA levels.	(27)
Rat	HFD, BCAA supplementation	BCAA supplementation caused the proliferation of R. *flavefaciens*, and the increased level of acetic acid subsequently inhibit lipogenesi-srelated genes expression, and avoid fat accumulation in the liver.	Increased R. *flavefaciens.*	Increased BCAA levels.	(30)
Mice	HFD, *bacteroides spp.* gavage	Supplementation of *Bacteroides spp.* reduced the defects in BCAA catabolism of BAT and avoid obesity in mice.	Supplementation of *Bacteroides spp.*	Reduced BCAA and BCKA levels in brown adipose tissue.	(31)
Human	T2DM patients	Intestinal dysbiosis characterized as an elevated abundance in *Prevotella copri*. was found in T2DM patients.	Increased *Prevotella copri* abundance in T2DM patients.	Increased serum BCAA levels.	(32)
Mice	HFD, PMFE gavage	PMFE gavage increased the abundance of commensal bacterium Bacteroides ovatus. In HFD mice, BCAA levels were decreased after gavaging with Bacteroides ovatus, and alleviated metabolic syndrome was relieved.	Increased abundance of *Bacterium Bacteroides ovatus*.	Decreased serum BCAA levels.	(33)
Human	Patients with lung cancer	The effect of gut microbiota on serum BCAAs concentration was prone to affected by the combinational influence of various bacteria, rather than individual microbial species. *Prevotella copri* and *Lactobacillus gasseri*. have positive correlation with BCAAs levels in non-cachectic patients.	*Prevotella copri* and *Lactobacillus gasseri* level increased in non-cachectic patients.	Increased serum BCAA levels in non-cachectic patients.	(34)
Mice	HFD, oral doses of ginsenoside Rb1 (200 mg/kg/day)	Rb1 supplementation decreased levels of BCAAs, and improved HFD induced insulin resistance.	19 genera showed strong correlation with serum BCAAs, Eubacterium corprostanoligenes was correlated with leucine and isoleucine simultaneously.	Rb1 supplementation decreased serum BCAA levels.	(35)
Mice	HFD, Prevotella *copri* gavage	The major bacteria that connect BCAA synthesis and insulin resistance are *Prevotella copri* and *bacteroides vulgatus.*	*Prevotella copri* gavage.	Increased circulating levels of BCAAs.	(28)
Mice	Normal diet, intermittent leucine-deficient food every other day	Leucine deficiency can change gut microbiome composition. Lack of leucine intermittently elevates *Bacteroidetes* and reduces *Firmicutes* at phylum level. Also, *Bacteroides, Alloprevotella and Rikenellaceae RC9* was elevated, *Lachnospiraceae* was reduced at genus level in leucine deficiency.	*Bacteroides, Alloprevotella and Rikenellaceae RC9* was increased, *Lachnospiraceae* was reduced.	Intermittent leucine deficiency.	(36)
Mice	Dietay supplementation BCAAem	The BCAAem supplemented group showed gut microbiota changes and lower serum concentrations of lipopolysaccharide-binding protein.	The abundance of *Akkermansia* and *Bifidobacterium* increased, *Enterobacteriaceae* decreased.	Increased BCAA levels.	(37)
Mice	HFD/STZ-induced T2DM mice, SF-Alg gavage	SF-Alg increased some benign bacteria, and decreased harmful bacteria. Meanwhile, SF-Alg dramatically decreased BCAAs in the colon of T2DM mice.	Beneficial bacteria such as *Lactobacillus*, *Bacteroides*, *Akkermansia Alloprevotella*, *Weissella* and *Enterorhabdus* were increased, deleterious bacteria (*Turicibacter* and *Helicobacter*) were decreased.	BCAAs decreased in the colon.	(38)
Mice	HFD, AB23A gavage	AB23A gavage decreased the abundance of the *Firmicutes*/*Bacteroidaeota* ratio and *Actinobacteriota*/*Bacteroidaeota* ratio, and significantly reduced serum lipopolysaccharide and BCAA levels.	*Firmicutes*/*Bacteroidaeota* ratio decreased and *Actinobacteriota*/*Bacteroidaeota* ratio decreased.	Decreased serum BCAA levels.	(39)
Rat	T2DM rats, SSJIBL	SSJIBL elevated the abundance of *Escherichia coli* and *Ruminococcus gnavus* in gut, and decreased *Prevotella copri* level, as well as the levels of serum BCAA.	Elevated abundance of gut *Escherichia coli* and *Ruminococcus gnavus*, reduction in *Prevotella copri*.	Decreased serum BCAA levels.	(40)
Human	Chronic haemodialysis patients, BCAA supplementation	The BCAA and glycine supplementation did not change faecal microbiota composition and microbial diversity, however, *L. paracasei* and *B. dentium* were reduced in abundance level.	Decreased abundance of *L. paracasei* and *B. dentium*.	Increased BCAA levels.	(41)

*HFD, high-fat diet; BCAA, branched-chain amino acid; DIO, diet-induce obesity; T2DM, type 2 diabetes mellitus; BCAAem, BCAA-enriched mixture; STZ, streptozotocin; SF-Alg, Sargassum fusiforme alginate; AB23A, Alisol B 23-acetate; SSJIBL, side-to-side jejunoileal bypass plus proximal loop ligation.*

## Role of Branched-Chain Amino Acids and Branched-Chain α-Keto Acid Dehydrogenase Kinase in Metabolic Disorders

### Obesity, Insulin Resistance, and Type 2 Diabetes Mellitus

Concentrations of plasma BCAAs, BCKAs, and carnitine esters, which derive from BCAAs metabolites are reported to be elevated in insulin resistance (IR), obesity, and type 2 diabetes mellitus (T2DM) patients and mice. And the circulating levels of BCAA and BCKA show an obvious correlation with body weight in mice as well as humans. Apart from the higher amount of food intake, the decreased metabolism of BCAAs contributes to another important aspect of obesity (42). It has been proven that the translation of BCAAs metabolism enzymes in adipose and liver is inhibited in obese mice, and the increased expression of BCKDK leads to the increased phosphorylation of BCKDH E1α and impaired activity of BCKDH in the liver (5, 43, 44). At the same time, BCKDK inhibitor treatment of diet-induced obese mice can inhibit weight gain and reduce the BCAAs and BCKAs concentrations in plasma (31). It is proposed that BCKDK promotes the occurrence of obesity by inhibiting BCAAs metabolism in obese animals. There are other studies pointing out that the elevated BCAAs in plasma might be related to impaired insulin action since it will increase protein catabolism in obese animals (31, 42). It is proposed that inflammation can down-regulate BCAA catabolism and lipogenesis in visceral adipose tissue partly through the NF-κB pathway, and endoplasmic reticulum stress can also down-regulate the BCAA metabolism pathway (45).

Muscle is reported as the earliest detectable tissue under insulin resistance abnormality, and the dysfunction of β-cell in the pancreas is widely accepted as the main pathophysiologic factor that drives T2DM (28, 46). It has been reported that elevated plasma BCAAs and BCKAs concentrations are early signs of insulin resistance in clinical studies (2). However, the level of BCAAs is not consistent with IR in different obesity-induced IR studies. In different tissues, the increase of circulating BCKAs is found to be better correlated with the severity of IR and T2DM, making BCKAs more effective and reliable biomarkers for IR (47–53). Accumulation of BCKAs is also an indicator of glucose intolerance and cardiac insufficiency and diabetes (54). The clearance of BCKAs is catalyzed by BCKDH, which is regulated by the inhibitory phosphorylation of BCKDK, suggesting BCKDK might be an important enzyme regulating this process.

BCAAs and BCKAs are reported to be involved in multiple IR pathways, including mTOR, insulin receptor substrate 1 (IRS1) pathway, fatty acid oxidation, and c-Jun NH2 terminal kinase (55–58). Activation of the mTORC1 pathway by leucine via Rag GTPases is considered to be an important mechanism of the impaired insulin signaling pathway. When activated, the tyrosine residues of insulin receptor substrates 1 and 2 (IRS1 and IRS2) were phosphorylated by insulin receptors, binding them to PI3K, causing AKT and protein kinase C (PKC) activated (59), those downstream effectors regulate glucose metabolism by phosphorylating downstream proteins in a cell-specific manner. AKT is considered a major and universal node in insulin signal transduction (60). It has been demonstrated that serum BCKAs inhibits insulin signaling by inhibiting AKT phosphorylation. Treatment with rapamycin, an mTOR inhibitor, showed a recovery in AKT2 phosphorylation and a moderate recovery of AKT1 phosphorylation suggesting that BCKAs inhibited AKT phosphorylation is dependent on mTOR (50, 59). Moreover, BCKAs have been proved to directly activate mTORC1, then activate S6K1 to phosphorylate the serine residue of insulin receptor substrate (IRS), preventing insulin receptors from binding to IRS and functioning (50).

Furthermore, it is proposed that activated mTORC1 can induce IR by directly promoting IRS1/2 degradation. However, in ob/ob mice given a low-protein diet, BCAAs supplementation causes insulin impairment without enhancing the activity of mTORC1. So that the activation of mTORC1 is not the only regulatory way of BCAA catabolism affecting insulin sensitivity (47). There are other ways leading to IR caused by BCAAs catabolic defects, such as increased synthesis and uptake of fatty acids due to BCAAs metabolic disorders. BCKDK can not only regulate BCAAs metabolism with BCAA as substrate but also phosphorylate ATP-Citrate Lyase (ACL), a critical enzyme in fatty acid synthesis from the beginning, and regulate the *de novo* formation pathway of fat, thus regulating the content of fatty acids (22). The metabolite of the valine, 3-HIB, may also cause insulin resistance through increasing muscle fatty acid usage (61). BCAAs supplementation to a normal diet does not affect mTOR activity while adding BCAAs to a high-fat diet increases mTOR activity in animal experiments. This proved that the existence of fatty acids promotes the activation of mTOR by BCAA and promotes the occurrence of IR. Abnormal BCAAs metabolism leads to the toxic stock of BCAAs metabolites, which triggers the dysfunction of mitochondria and stress signaling related to insulin resistance and T2DM. An example is JNK, which is associated with insulin resistance (62, 63).

### Cardiovascular Disease

As mentioned above, BCAAs are found to be increased in the failing heart. It is proposed that cardiac BCAAs oxidation is decreased in insulin resistance. A study showed that BCAAs oxidation dedicated about one percentage of ATP production in heart ATP production, however, the main sources of heart ATP production are glucose and fatty acid oxidation. This suggests that cardiac insulin resistance is not due to the increased oxidation of BCAAs, which inhibits glucose and fatty acid oxidation (64). However, it is not clear whether BCAAs result in heart failure and mediate cardiac insulin resistance. In a permanent myocardial infarction (MI) model, utilizing coronary artery ligation, BCAAs catabolism was damaged seriously in the myocardium. This leads to an obvious elevation in BCAAs levels and activates mTOR signaling, exacerbating cardiac dysfunction and remodeling (65). It has been reported that BCAA levels were increased in dilated cardiomyopathy (DCM) hearts, accompanied by a decreased expression of mitochondrial BCAT2 and total expression of BCKDH compared to non-failing control. Also, phosphorylation of BCKDH and expression of cardiac PP2Cm were reduced in the DCM hearts, with an unchanged expression of BCKDK. It is reported that in heart failure patients, KLF15 expression is inhibited through the TAK1/P38MAPK axis, thus inhibiting BCAA catabolism, which leads to BCAAs accumulated in the heart. Also, increasing the oxidation rate of BCAAs has proved a practicable way to improve the contractile function of failing hearts in mice. After injecting BCKDK inhibitor, there is an increase in the activity of BCKDH and oxidation of BCAAs and the% ejection fraction (%EF) of TAC mouse increased significantly in transverse aortic constriction (TAC) surgery as heart failure mouse models. The results suggest that lowering cardiac BCAA levels by increasing the BCAA oxidation rate might offer a novel therapeutic way of heart failure treatment (66).

### Exercise Damage

BCAAs are believed to increase muscle mass and limit exercise damage, so they are often utilized as nutritional supplements after exercise by some athletes and people with exercise habits regardless of their practice level. To investigate whether BCAAs supplements are beneficial, 11 studies described tests performed on humans who took BCAAs supplementation orally as a nutritional strategy were included, and a systematic review was published by some researchers, providing a comprehensive analysis of the effects of BCAAs supplementation on muscle damage induced by exercise. The result is quite neutral because the positive effect study numbers was equal to the no effect ones. What’s more, the studies which reported BCAAs supplementation had a positive effect on muscle damage, appeared to be of low quality. Most of the high-quality studies suggest there is no significant effect of BCAAs supplementation. This systematic review proposed that whether BCAAs supplementation is beneficial in muscle damage is uncertain. But under certain circumstances, supplying BCAAs might lessen the exercise-induced muscle damage. Under these circumstances, the experiment subjects are asked to take BCAAs supplementation at a dosage greater than 200 mg/kg/day, starting 1 week before exercise, and with a time duration longer than 10 days (67). To investigate whether BCAAs supplementation is needed to prevent exercise-induced muscle damage, there are more experiments with a larger sample size that needs to be done in the future.

### Nervous System Diseases

As an important subgroup of essential amino acids, BCAAs in particular leucine, play an important role in the stimulation of protein synthesis, and cellular signaling. Some nervous system diseases are found to be related to be lack of BCAAs caused by mutations in the gene *BCKDK*. In a colony of Sprague–Dawley rats, there is a new mutant with the characteristics of hindlimb splaying, seizures, and brain weight decreasing. Mutations in the gene *Bckdk* are found in those rats (68). A study first reported that *Bckdk*^–/–^ mice displayed repetitive hind limb flexion and extension when hung by the tail and eventually developed epileptic seizures (69). By studying those consanguineous families with autism, epilepsy, and intellectual disability, researchers have found mutations in the inactivating gene *BCKDK*. Patients with homozygous *BCKDK* mutations show decreasing messenger RNA and protein of *BCKDK* and plasma BCAAs. *Bckdk* knock-out mice have abnormal amino acid profiles in the brain and neurobehavioral deficits that respond to dietary BCAA supplementation (70). Another study discovered two novel mutations in the gene *BCKDK* of two children who are not related to each other, and their body fluids’ BCAA levels were decreased. Also, their delay in development and abnormal neurobehaviors were partially corrected after BCAA supplementation in a rich-protein diet (71).

Also, *BCKDK* and BCAAs are found to be related to energy metabolism in Huntington’s disease, which has the characteristics of progressive dysfunction in activity and cognition, with the symptoms of speech cessation, swallowing problems, walking difficulties, and losing independence, and dementia attack in the end. By measuring the blood of Huntington’s disease gene carriers and healthy controls in metabolites and gene expression changes, 6 statistically significant mRNA transcripts were found, and *BCKDK* was 1.34-fold higher in gene transcription level than healthy controls (72).

In maple syrup urine disease (MSUD) patients, accumulation of BCAAs and BCKAs are found due to the deficiency of branched-chain α-keto acid dehydrogenase caused by some certain inborn error of metabolism. It is proposed that the toxic accumulation of BCAAs and BCKAs can lead to apoptosis in glial cells and neurons with dosage and time-dependent pattern (73). Destroying the metabolism of brain energy is another mechanism. Pyruvate dehydrogenase, α-ketoglutarate dehydrogenase and the mitochondrial respiration chain are all inhibited by the accumulating BCKAs and leucine (74).

### Treatment Targeting Branched-Chain α-Keto Acid Dehydrogenase Kinase and Branched-Chain Amino Acids

The therapeutic way of treating BCKDK deficiency is to restore the normal BCAA levels in patient fluids currently (75). The supplementation of BCAAs is quite important in nervous system diseases with mutations in the gene *BCKDK*. Nowadays, the research focus is mainly on decreasing the circulation BCAAs by inhibiting BCKDK or other methods.

The mitochondrial BCKDH is negatively regulated by reversible phosphorylation, and recent studies have found a novel BCKDK inhibitor, compound 3,6-dichlorobenzo[b]thiophene-2-carboxylic acid (BT2) can reduce the concentrations of BCAA *in vivo*. BT2 binds to BCKDK and leads to helix movements in the N-terminal domain, causing the dissociation of BCKDK from the BCKDH along with accelerated degradation of the released kinase *in vivo* (54, 76). When DIO mice were administered BT2 by oral gavage, serum BCAA and BCKA concentrations in the BT2 group showed a remarkable reduction compared with those in the control group after 4 weeks of BT2 treatment. Although the food intake amount of a high-fat diet appeared no difference in the two groups, BT2 treatment can obviously inhibit the weight gain compared with control (31). It is proposed that when wild-type mice were treated with BT2 at a dosage of 20 mg/kg/day for 1 week, it will result in almost completely dephosphorylated and maximally activated BCKDH in the heart, muscle, kidneys, and liver with a decrease in serum BCAA concentrations (76).

Similar to BT2, another BCKDK inhibitor (s)-α-chloro-phenylpropionic acid [(S)-CPP] is believed to achieve the same effect of reducing plasma BCAA concentrations. (S)-CPP binds to the N-terminal domain in a unique allosteric site, causing helix movements in BCKDK. These conformational changes are transferred to the lipoyl-binding pocket, which prevents BCKDK from binding to the BCKDH core to remove its activity (77).

There is also an easily available drug in the clinic found useful in improving BCAAs catabolism. Pyridostigmine has been widely used and tested in the clinic for the treatment of myasthenia gravis. Recently, some studies have revealed that pyridostigmine inhibits cholinesterase to increase acetylcholine levels, leading to an improvement in cardiac function in rats with cardiovascular diseases. At the same time, the cardiac function of normal rats was not affected. Also, pyridostigmine was found to promote BCAAs catabolism by enhancing vagal activity and attenuating intestinal barrier injury and gut bacteria dysbiosis in diabetic cardiomyopathy mice. Pyridostigmine was also found to enhance cardiac BCAAs catabolism by upregulating BCAT2 and PP2Cm and downregulating p-BCKDHA/BCKDHA and BCKDK (78).

Nowadays, insulin sensitizer therapy such as thiazolidinediones is found to improve insulin sensitivity and reduce serum BCAAs (79–81). A study reported that thiazolidinediones (in this experiment, rosiglitazone was used) treated mice had lower circulating BCAA and showed upregulated BCAT as well as BCKDH activities compared with those treated with placebo, independently of diet. They hypothesized that rosiglitazone could reduce IRS1 serine phosphorylation in skeletal muscle dependent on mTORC1 and thus reduce insulin resistance throughout the body (79).

## Conclusion

**FIGURE 3:**
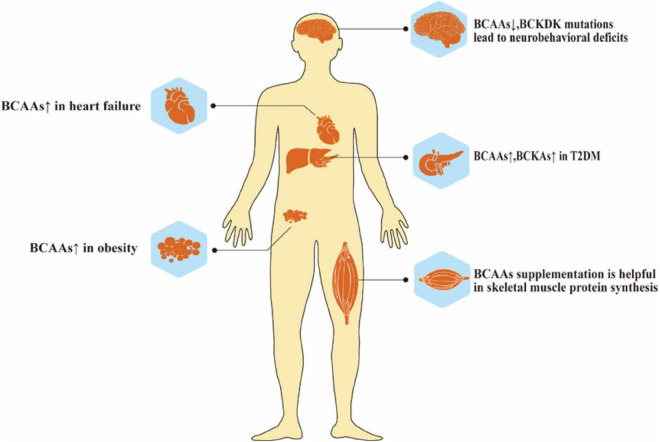
Role of BCAAs metabolism in health. Abnormal elevation or deficiency of BCAAs lead to many diseases, such as neurobehavioral deficits, heart failure, T2DM and obesity.

The homeostasis of BCAAs is critical in health and disease. Like essential amino acids, BCAAs participate in protein synthesis, regulating neurotransmitter synthesis, providing energy support, and acting as nitrogen donors. The accumulation and insufficiency of BCAAs play critical roles in different metabolic disorders (Figure 3). Lacking BCAAs and BCKDK mutations may cause severe neurological disorders and growth retardation. However, insulin resistance, obesity, heart failure, and even cancer are all associated with the accumulation of excess BCAAs. BCKDK, as an important enzyme affecting the metabolism of BCAA, is now recognized as a new therapeutic target for treating metabolic disorders caused by BCAA accumulation. Certain microbiota may also affect BCAA metabolism, and further studies are needed in this area. Apart from introducing diseases related to BCAAs and BCKDK, this review has also offered information about some novel detection methods of BCAAs, the interaction between BCAAs and microbiota, and BCKDK inhibitors, facilitating the researchers to get a comprehensive understanding of studies on BCAAs.

## Author Contributions

All authors listed have made a substantial, direct, and intellectual contribution to the work, and approved it for publication.

## Conflict of Interest

The authors declare that the research was conducted in the absence of any commercial or financial relationships that could be construed as a potential conflict of interest.

## Publisher’s Note

All claims expressed in this article are solely those of the authors and do not necessarily represent those of their affiliated organizations, or those of the publisher, the editors and the reviewers. Any product that may be evaluated in this article, or claim that may be made by its manufacturer, is not guaranteed or endorsed by the publisher.
